# Predicting Pulmonary Exacerbations in Cystic Fibrosis Using Inflammation-Based Scoring Systems

**DOI:** 10.3390/diagnostics15212761

**Published:** 2025-10-31

**Authors:** Raphael S. Reitmeier, Melanie Götschke, Julia Walter, Jeremias Götschke, Julian Schlatzer, Diego Kauffmann-Guerrero, Jürgen Behr, Amanda Tufman, Pontus Mertsch

**Affiliations:** 1 Department of Medicine V, LMU University Hospital, LMU Munich, 81377 Munich, Germany; 2 Comprehensive Pneumology Center (CPC-M), Member of the German Center for Lung Research (DZL), 81377 Munich, Germany

**Keywords:** cystic fibrosis, CF, biomarkers, inflammation, inflammation-based scoring, pulmonary exacerbations

## Abstract

**Background**: The aim of this study is to identify people with cystic fibrosis (pwCF) at risk for future pulmonary exacerbations (PEx) based on established and unestablished markers of chronic inflammation. There is currently no universal definition of PEx in cystic fibrosis (CF), but it is commonly characterized by clinical deterioration and a drop in FEV1 ≥10% with or without elevations in systemic inflammatory markers. PEx negatively affect clinical outcomes in pwCF; therefore, predicting and preventing PEx is a crucial goal in the treatment of pwCF. **Methods**: We retrospectively examined pwCF ≥18 years who had ≥2 pulmonary function tests per year for a 3-year period. The first year was marked as the baseline. The follow-up period (FU) was defined as the following two-year period after baseline. PEx were defined as a need for intravenous antibiotic treatment due to clinical deterioration. Various scoring systems and ratios (neutrophil/lymphocyte (NLR), lymphocyte/monocyte (LMR), CRP, CRP/albumin, Glasgow Prognostic Score (GPS), high-sensitivity modified Glasgow Prognostic Score (hs-GPS)) were compared in pwCF with and without PEx during the FU. Logistic regression models were used to determine the best marker for predicting PEx, considering factors such as age, sex, PEx at baseline, BMI, homozygote F508del mutation, diabetes mellitus, chronic bacterial infection, and CFTR (cystic fibrosis transmembrane conductance regulator)-modulator therapy. The results are reported as odds ratios (ORs) with *p*-values. **Results**: Out of 283 pwCF, 131 were included in the study. In total, 43.5% were female, and the mean age was 34.0 years. A total of 75 pwCF (57.3%) had PEx during FU. In the multivariate analysis, the following markers at baseline were significantly associated with having a PEx during FU: CRP(log) (OR = 7.29, *p* = 0.01), CRP/albumin (OR = 1.08, *p* = 0.006), decreased LMR (OR = 0.51, *p* = 0.02), increased NLR (OR = 1.52, *p* = 0.02), and GPS of 1 vs. 0 (OR = 2.75, *p* = 0.04). The results indicate that the CRP/albumin ratio was the best model for predicting PEx in pwCF during the FU, outperforming other models. **Conclusions**: While several inflammation-based scoring systems can predict PEx in pwCF, the easily calculated CRP/albumin proved to reliably identify pwCF with an increased risk for PEx, making it a promising tool in clinical practice.

## 1. Introduction

Cystic fibrosis (CF) is an autosomal recessive disease caused by different mutations in the cystic fibrosis transmembrane conductance regulator (CFTR) gene. It is the second most common autosomal recessive disease in Germany and is the most common genetic disease leading to death [1,2]. With the introduction of CFTR modulators, the median life expectancy of affected individuals has increased to 66.8 years, though it is still life-limiting [3]. In most people with CF (pwCF), pulmonary disease is the leading clinical feature, with repeated acute pulmonary exacerbations (PEx) and mortality. PEx are usually caused by bacterial and viral infections, with pulmonary colonization with *Pseudomonas aeruginosa* (PA) and other pathogens being characteristic [4].

PEx are often associated with a permanent loss of lung function [5,6]. Identifying pwCF at an increased risk for frequent PEx in order to prevent a worse outcome is an important treatment goal. The optimal prediction of PEx should be achievable through widely available and easily accessible diagnostic tests, potentially utilizing either a single parameter or a combination of parameters. Scoring systems for CF already exist, including scores for the current disease severity, short-term intervention prognosis, future risk of lung transplantation, and indication of current PEx [7].

Inflammation, especially chronic neutrophilic inflammation, plays a key role in the pathogenesis of CF and is associated with lung function decline, structural airway remodeling, and the development of bronchiectasis [8,9,10]. This knowledge and the fact that many established scoring systems for CF already include inflammation-based parameters highlight the potential of inflammation-based scoring systems and ratios as predictors for future PEx risk [7]. However, there is limited evidence on the utility of inflammation-based scoring systems in CF, especially in adult pwCF. Existing studies have primarily focused on pediatric populations and have yielded conflicting results [11,12,13,14]. Given that disease severity and inflammatory burden tend to be greater in adults, inflammation-based scoring systems may prove more effective in adult CF populations.

The goal of our work is to evaluate the differential utility of established inflammation-based markers in relation to PEx frequency in order to improve targeted, specialized care.

## 2. Materials and Methods

**Figure 1:**
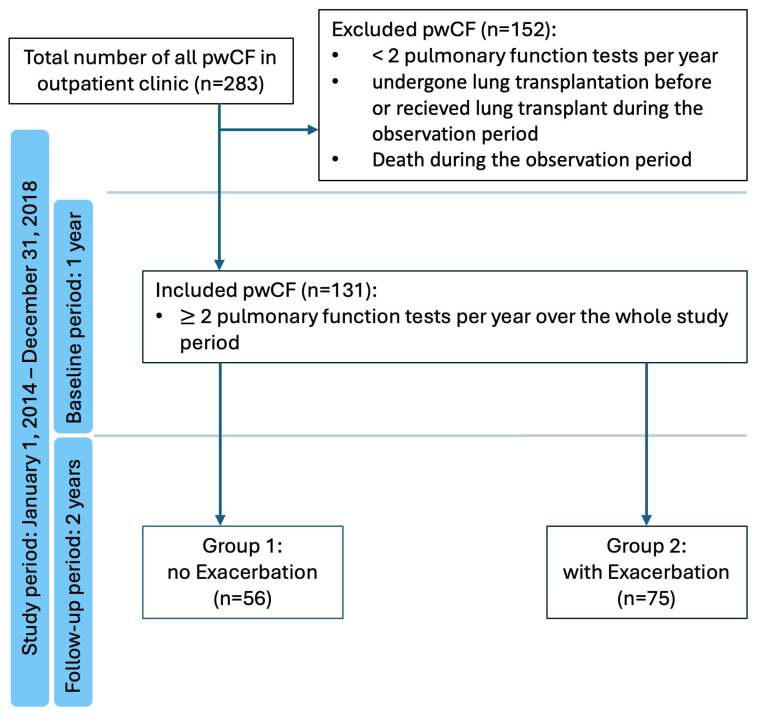
Flowchart of the chronological course of data analysis with exclusion and inclusion criteria.

In this retrospective study, we included all pwCF aged 18 or older treated in the CF unit at the University Hospital of the LMU, Munich, between 1 January 2014 and 31 December 2018. PwCF undergoing pulmonary function testing at least twice a year for 3 years during the observation period were eligible for evaluation (Figure 1). Approval for the study was obtained from the responsible Ethics Committee (Reference number 19-645).

### 2.1. Data Collection

Clinical data (sex, age, height, body weight, mutation status, concomitant diseases, information on antibiotic therapy, medication) were collected from medical records for all routine outpatient visits during the three-year study period. Body mass index (BMI) was calculated from age and height. The following lab results were collected: CRP (mg/dL) and albumin (g/dL). Additionally, results of the blood count and, whenever available, the differential blood count were included: Leukocytes lymphocytes (G/L), lymphocytes (%), monocytes (G/L), and monocytes (%). The parameters FEV1 and FEV1% of the body plethysmography were collected from clinical information systems. Furthermore, the results of microbiological examinations were collected, obtained from bronchoalveolar lavage (BAL) and sputum. The evaluation was semiquantitative.

### 2.2. Defintion of PEx

The primary endpoint of the study was the occurrence of PEx, defined as a clinical deterioration requiring intravenous antibiotic therapy. This usually reflected an acute PEx, although in some pwCF with chronically impaired health status, intravenous antibiotic therapy could be initiated preemptively to prevent further decline before marked acute worsening occurred during the follow-up period (FU). The need for intravenous antibiotic therapy was a clinical decision by the treating physician.

### 2.3. Scoring Systems

The inflammation-based prognostic score known as the Glasgow Prognostic Score (GPS), defined by elevated serum C-reactive protein (CRP) and reduced albumin levels, is the most validated inflammatory risk marker in oncology, with a possible score range of 0–2 points [15,16]. The high-sensitivity modified Glasgow Prognostic Score (hs-GPS), which utilizes a more stringent cutoff for CRP levels, has been reported to serve as a superior prognostic indicator for various malignancies [17,18]. The scores were used throughout the entire enrollment period. The neurophil–lymphocyte ratio (NLR) was calculated by dividing the value of lymphocytes by the value of neutrophils. The identical procedure was used to calculate the lymphocyte–monocyte ratio (LMR) and the CRP/albumin ratio.

### 2.4. Statistical Analysis

The observation period was divided into two parts: baseline and FU. Baseline was defined as the first year of the observation period, and the subsequent period was classified as FU. PwCF were divided into two groups: one group with PEx in the FU and one group without PEx in the FU. We reported categorical variables as absolute and relative frequencies and numerical variables as means with standard deviation (sd). We used Chi^2^-test, Fischer exact test, and Student’s *t*-test in univariate analysis to compare categorical and numerical values, respectively. In the multivariate analysis, logistic regression models with log link were used adjusted for age, sex, PEx at baseline, BMI, homozygous F508del mutation, diabetes mellitus, chronic lung infections (*Pseudomonas aeruginosa*, *Stenotrophomonas maltophilia*, *Mycobacterium abscessus*, *Achromobacter xylosoxidans*), and use of a CFTR modulator. Because CRP showed a right-skewed distribution, we examined whether its relationship with the outcome was linear. Both a Box–Tidwell test (*p* = 0.43) and visual inspection suggested approximate linearity of the logit for log-transformed CRP. A natural cubic spline indicated mild nonlinearity (*p* = 0.012), which was largely resolved after log transformation. Therefore, log (CRP) (CRP (log)) was used in the final model to improve interpretability and model fit. Odds ratios (ORs) for CRP (log) thus represent the change in odds of PEx per one-unit increase in log (CRP), corresponding approximately to a 2.7-fold increase in raw CRP concentration. Other laboratory parameters were entered on their original scales. For the CRP/albumin ratio, CRP was used on the original (non-log-transformed) scale, and the variable was multiplied by 100 to facilitate interpretation; ORs are expressed per one-unit increase in this scaled ratio. For all other continuous variables, ORs reflect the change in odds per one-unit increase in the respective variable. The Akaike information criterion (AIC) of the models were compared to determine the marker with the best predictive power. Results from logistic regression analysis are expressed as ORs with *p*-values. Statistical significance was determined using *p*-values with alpha errors <0.05. We used R version 4.5.1 with RStudio Version 2025.05.1 + 513 to perform all statistical analysis. Tables were created in Microsoft Excel.

## 3. Results

### 3.1. Cohort Characteristics

Among the 283 pwCF treated at our CF outpatient clinic between January 2014 and December 2018, 131 met the inclusion criteria and had an FU with at least two pulmonary function tests per year. Of these, 43.5% were female, with a mean age of 34 years (range: 18–61 years). Additionally, 53.4% of pwC were homozygous for the F508del mutation. During the baseline period, 64 pwCF (48.9%) experienced at least one PEx. Throughout the 2-year FU, 75 (57.3%) of the pwCF experienced at least one PEx, with a mean PEx rate of 1.4 (SD ± 1.9). A significant predictor of recurrent PEx during FU was a history of PEx in the baseline period, with 52 pwCF (69.3%) experiencing another PEx. A higher BMI within the normal range was associated with a significantly lower incidence of PEx. Interestingly, pwCF undergoing CFTR modulator therapy exhibited a higher frequency of PEx. Table 1 displays characteristics stratified by PEx during FU.

### 3.2. Marker and Scores

Table 2 presents the markers and scoring systems evaluated for their ability to predict PEx during the FU.

#### 3.2.1. CRP

The log-transformed serum CRP was significantly higher in the PEx group compared to the group without PEx. In the multivariate analysis, the CRP(log) at inclusion was found to be significantly associated with the likelihood of experiencing PEx, with an OR of 2.23 (*p* = 0.004). This corresponds to a 2.23-fold increase in the odds of PEx for each one-unit increase in CRP (log), which is equivalent to approximately a 2.7-fold rise in raw CRP concentration.

#### 3.2.2. Albumin

The mean serum albumin was 4.26 ± 0.31 g/dL and was lower in pwCF with PEx than in those without (4.19 ± 0.30 vs. 4.35 ± 0.29 g/dL, *p* < 0.0001). Although not statistically significant higher, albumin was associated with reduced odds of PEx (OR 0.35, *p* = 0.21).

#### 3.2.3. CRP/Albumin

**Figure 2:**
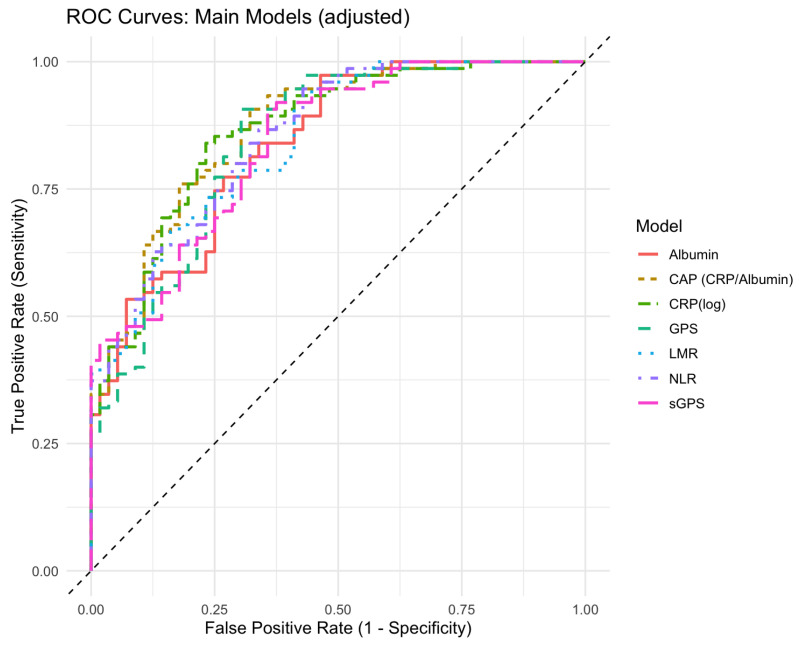
ROC curves for the main prognostic models: albumin, CRP/albumin ratio (CAP), log-transformed CRP (CRPlog), Glasgow Prognostic Score, high-sensitivity Glasgow Prognostic Score (sGPS), neutrophil-to-lymphocyte ratio, and lymphocyte-to-monocyte ratio. Optimal cut-off values were determined using Youden’s index from the ROC analysis. ROC: receiver operating characteristic; AUC: area under the curve. GPS, Glasgow Prognostic Score; sGPS, high-sensitivity Glasgow Prognostic Score (hs-GPS); LMR, lymphocyte–monocyte ratio; NLR, neutrophile–lymphocyte ratio; CRP, C-reactive protein.

The mean CRP/albumin ratio was significantly higher in the PEx group (0.25 ± 0.32 vs. 0.07 ± 0.06, *p* < 0.0001). Logistic regression showed that an increase in the ratio was associated with a higher likelihood of having PEx in the FU (OR 1.08, *p*= 0.006). The AIC of this model was 140.1. The optimal cut-off value for the ratio was 0.42, with a specificity of 0.68 and a sensitivity of 0.91; the AUC was 0.87 (Figure 2).

#### 3.2.4. LMR

The mean LMR of all pwCF was 3.11 ± 1.90. In pwCF with PEx, the average score was lower compared to those without PEx (2.71 ± 0.96 vs. 3.65 ± 2.61, *p* < 0.0001). A higher LMR was significantly associated with reduced odds of PEx (OR 0.51, *p* = 0.018).

#### 3.2.5. NLR

The NLR of all pwCF averaged 4.03 ± 2.53. In pwCF with PEx, the average was 4.83 ± 2.88, and in pwCF without PEx the average was 2.98 ± 1.45 (*p* < 0.0001). A higher NLR was significantly associated with a higher risk of having PEx during FU (OR 1.52, *p* = 0.015).

#### 3.2.6. GPS

At baseline, 63.4% of the 131 pwCF had a GPS of 0, 30.5% had a score of 1, and 6.1% had a score of 2. Among pwCF with PEx (n = 75), 49.3% had a score of 0, 40.0% a score of 1, and 10.7% a score of 2. In the group without PEx, 82.1% had a score of 0 and 17.9% a score of 1. No pwCF without PEx had a score of 2. A GPS of 1 was significantly associated with PEx during FU compared to a GPS of 0 (OR 2.75, *p* = 0.038).

#### 3.2.7. hs-GPS

At baseline, 27.5% of the 131 pwCF had an hs-GPS of 0, 65.6% had a score of 1, and 6.9% had a score of 2. Among pwCF with PEx (n = 75), 17.3% had a score of 0, 72.0% a score of 1, and 10.7% a score of 2. In the group without PEx, 41.1% had a score of 0, 57.1% had a score of 1, and 1.8% had a score of 2. An hs-GPS of 1 was not associated with PEx during FU compared to an hs-GPS of 0 (OR 2.17, *p* = 0.093).

#### 3.2.8. Model Comparison

The predictive performance of the logistic regression models of the used scoring systems for future PEx, were compared using the AIC. Lower AIC values indicate a better model fit, while accounting for model complexity. Among the models tested, the CRP (log)-based model (AIC = 143.8) and the CRP/albumin ratio model (AIC = 140.1) demonstrated a superior fit in predicting PEx. CRP-based indices provided the best performance in predicting PEx. Interestingly, the GPS and hs-GPS, which rely on fixed cut-offs for CRP and albumin, showed inferior performance in predicting PEx compared to CRP/albumin and CRP (log) comparing AIC and AUC. Similarly, the cell-based ratios LMR and NLR demonstrated a lower predictive value. Table 3 and Table 4 show the results of the logistic regression and model comparison. Details on the full logistic regression analysis can be found in the Supplementary Materials (Table S1). Stratified analyses by baseline PEx history showed similar trends, with the strongest discrimination observed in pwCF without prior PEx (AUCs up to 0.90). In contrast, among those with previous events, estimates were directionally similar but not statistically significant, likely reflecting a smaller subgroup size and maybe a more inflamed phenotype. These results suggest that the assessed biomarkers may be most informative in identifying pwCF at risk before clinical deterioration becomes clinically apparent (Tables S2–S5).

## 4. Discussion

In this retrospective study, we aimed to identify simple and easy-to-acquire markers that can predict future PEx in pwCF. We were able to show that various scores and ratios provide an indication of the risk for future PEx. The logistic regression analysis showed significant results for CRP(log), CRP/albumin, NLR, LMR, and GPS, but not for albumin and hs-GPS, according to Table 3. In a comparison of the scores and ratios examined, we found that CRP/albumin and CRP performed better at predicting future PEx compared to other inflammation scores.

GPS, LMR, and NLR were originally developed for estimating the prognosis of malignant diseases [16,19,20]. Interestingly, they also showed significant results in our CF cohort. Pronounced neutrophilic inflammation is associated with cancer progression and worse outcomes in cancer [21]. It also plays a crucial role in CF development and disease progression, with elevated neutrophils being associated with worse outcomes [8,22,23]. As systemic inflammation forms the basis of most scoring systems evaluated here, this may explain their applicability across both malignant conditions and CF.

The fact that systemic neutrophil-driven inflammation is a feature across different CF genotypes is also evident in our study, as only 53.4% of the studied CF population were F508del homozygous. This supports the relevance of the assessed scoring systems in genetically diverse CF populations as potentially useful tools for assessing the future risks of PEx.

Due to their simplicity and broad availability, serum CRP levels are an interesting marker in clinical routines. We found that the median serum CRP level was significantly higher in the PEx group. Furthermore, our multivariate analysis revealed a significant association between CRP levels at inclusion and the likelihood of experiencing PEx during FU. In previous studies, CRP consistently correlated with disease activity [24]. Our results provide supporting evidence that serum CRP levels may serve as a simple, clinical valuable predictive marker for identifying pwCF at increased risk of PEx. Apart from that, previous PEx were also significantly associated with future PEx. This is in line with previous research, which also showed that having at least one intravenously treated PEx in the previous year is an independent risk factor for future PEx in pwCF [25]. It is important to note that, even after adjusting for previous PEx, we were able to show in the study that the CRP/albumin ratio had a significant correlation with future PEx. At a threshold of 0.42, the CRP/albumin ratio showed a high sensitivity (91%) with moderate specificity (68%). In our cohort, this would mean that approximately 18 out of 56 pwCF without PEx would be incorrectly classified as high risk. For these pwCF, this could potentially lead to unnecessary treatment or increased anxiety due to concerns about disease worsening. However, since CRP and albumin are routinely measured during regular outpatient visits, the CRP/albumin ratio provides a simple, cost-neutral, and readily available tool to help identify pwCF at an increased risk of PEx.

In our adult CF population, NLR was significantly higher in pwCF with PEx. Previous studies in pediatric cohorts have evaluated NLR with mixed results. A retrospective study of 159 children reported that NLR ≥ 3 was associated with lower BMI and ppFEV1 but did not assess PEx risk [11]. In a newborn screening cohort, NLR showed no correlation with weight or height [12]. Tung et al. found higher NLR values during clinical stability in 141 children, especially in females and with increasing age, but no association with lung function [14]. None of the pediatric studies assessed NLR as a predictor of future PEx. A Brazilian study of 38 children/adolescents found that an elevated CRP/albumin ratio predicted lower lung function after three years [13]. These findings suggest that the prognostic value of inflammation-based scores may be more pronounced in adults with more advanced disease, as seen in our study.

This study has several limitations. In addition to its retrospective and monocentric design, there is a potential for selection bias, since only pwCF with regular FU were included. This may have excluded pwCF who either attended routine visits irregularly due to good health, were too ill to participate in FU assessments, underwent lung transplantation, or died during the study period. Consequently, the presence of a survival bias cannot be ruled out, although pwCF with irregular attendance due to good health status were also excluded. Furthermore, intravenous antibiotic treatments due to clinical deterioration were used as a surrogate marker for PEx. While consequent outpatient intravenous treatments were included, treatment with oral antibiotics for milder PEx was not captured, which likely led to an underestimation of overall PEx frequency. It is also possible that some pwCF classified as having no PEx may in fact have experienced milder events, and therefore the groups may not be completely homogenous. On the other hand, we focused exclusively on intravenously treated PEx, which resulted in a more homogeneous group, as intravenous therapy typically reflects a higher threshold of clinical deterioration and disease severity before being initiated. Similarly, limiting the cohort to pwCF with consistent FU and documented intravenous treatments can be considered a strength of the study, as it ensures a more uniform and clinically well-characterized study population.

The use of CFTR modulators, which improve lung function and reduce PEx rates in pwCF, may have influenced our findings. As therapy use varied within the cohort, potential confounding cannot be excluded. Since, during the time of observation, only first-generation modulators (ivacaftor, lumacaftor/ivacaftor, tezacaftor/ivacaftor) with a limited efficacy compared to the now available highly effective modulator therapy (e.g., elexacaftor/tezacaftor/ivacaftor, vanzacaftor/tezacaftor/deutivacaftor,) were licensed, the overall impact is likely minor. This is supported by the higher proportion of modulator-treated individuals in the exacerbator group, reflecting clinical practice where therapy was often initiated in pwCF with more severe disease, introducing a potential indication bias. The observed association between modulator use and higher PEx rates likely reflects underlying disease severity and should be interpreted in this context. In a sensitivity analysis excluding pwCF receiving CFTR modulator therapy, CRP, CRP/albumin, and LMR remained significant predictors of future PEx, indicating that the prognostic value of inflammatory biomarkers persisted independent of modulator use (Tables S6 and S7). As the landscape of CF treatment has changed substantially since the study period (2014–2018), with the widespread introduction of highly effective CFTR modulators and a marked reduction in PEx [26], the generalizability of these findings to current clinical practice may be limited. Future studies should validate the performance of the tested biomarkers in pwCF receiving modern highly effective modulator treatment.

As logistic regression was applied, the analysis was limited to a binary outcome representing the occurrence of at least one PEx during FU. This approach does not consider when or how often PEx occurred and may therefore miss time-related patterns. In addition, it does not account for the fact that some pwCF experienced multiple events. While logistic regression provides an interpretable way to examine biomarker associations, it simplifies a complex longitudinal process.

## 5. Conclusions

This study demonstrates that inflammation-based biomarkers, well-established in oncology, may also serve as useful predictors of clinical deterioration in pwCF. Specifically, elevated CRP levels and the CRP/albumin ratio were most strongly associated with an increased risk of PEx. These markers are routinely available in clinical practice and may aid in the early identification of high-risk pwCF who could benefit from closer monitoring and intensified or earlier treatment. While the retrospective design and limited sample size represent important limitations, these results should be regarded as hypothesis-generating and require confirmation in larger, prospective cohorts before translation into routine clinical practice. Additionally, ongoing shifts in CF care with the broad use of highly effective CFTR modulator therapies must be considered for future research. Investigating the predictive value of these markers in modulator-treated populations might help to identify pwCF with an increased risk for clinical deterioration. Overall, our findings highlight the potential role of simple inflammatory markers in improving individualized risk assessment and guiding clinical decision-making in the management of pwCF.

## Figures and Tables

**Table 1:** Baseline characteristics grouped by exacerbation status during follow-up.

	All pwCF (n = 131)		With Exacerbations (n = 75)		Without Exacerbations (n = 56)		
	Mean	SD	Mean	SD	Mean	SD	*p*-Value
Age	34	9	33.7	8.9	34.5	9.2	0.6
BMI	21.7	2.8	20.8	2.2	22.8	3.1	<0.0001
All pwCF (n = 131)	n	%	n	%	n	%	*p*-value
ppFEV1							<0.0001
>80%	44	33.6	13	17.3	31	55.4	
79–50%	46	35.1	27	36.0	19	33.9	
49–30%	35	26.7	29	38.7	6	10.7	
<30%	6	4.6	6	8.0	0	0.0	
Male sex	74	56.5	41	54.7	33	58.9	0.76
Diabetes mellitus	46	35.1	31	41.3	15	26.8	0.12
delF508 homocygote	70	53.4	42	56.0	28	50.0	0.61
CFTR modulator therapy	20	15.3	16	21.3	4	7.1	0.03
Microbiology	n	%	n	%	n	%	*p*-value
*Pseudomonas aeruginoas*	97	74.0	60	80.0	37	66.1	0.11
*Stenotrophomonas maltophilia*	25	19.1	18	24.0	7	12.5	0.15
*Mycobacterium abscessus*	6	4.6	5	6.7	1	1.8	0.24
*Achromobacter xylosoxidans*	14	10.7	11	14.7	3	5.4	0.16
Exacerbations	n	%	n	%	n	%	*p*-value
During baseline period	64	48.9	52	69.3	12	21.4	<0.0001

BMI, body mass index; CFTR, cystic fibrosis transmembrane conductance regulator; pwCF, people with cystic fibrosis.

**Table 2:** Different scores, ratios, and parameter categorized for all pwCF, as well as stratified by presence or absence of exacerbations during follow-up period.

		All pwCF (n = 131)		With Exacerbations (n = 75)		Without Exacerbations (n = 56)		
		n	%	n	%	n	%	*p*-Value
GPS	0	83	63.4	37	49.3	46	82.1	
	1	40	30.5	30	40.0	10	17.9	
	2	8	6.1	8	10.7	0	0.0	0.0001
hs-GPS	0	36	27.5	13	17.3	23	41.1	
	1	86	65.6	54	72.0	32	57.1	
	2	9	6.9	8	10.7	1	1.8	0.003
		mean	sd	mean	sd	mean	sd	
Albumin		4.26	0.31	4.19	0.3	4.35	0.29	<0.0001
CRP(log) [median, IQR]		0.35	0.17; 0.82	0.61	0.24; 1.36	0.21	0.12; 0.43	<0.0001
CRP/albumin		0.18	0.26	0.25	0.32	0.07	0.06	<0.0001 *
LMR		3.11	1.9	2.71	0.96	3.65	2.61	<0.0001
NLR		4.10	2.54	4.87	2.88	3.04	1.44	<0.0001

GPS, Glasgow Prognostic Score; hs-GPS, high-sensitivity Glasgow Prognostic Score; LMR, lymphocyte–monocyte ratio; NLR, neutrophile–lymphocyte ratio; IQR, interquartile range; pwCF, people with cystic fibrosis; * Wilcoxon rank sum test.

**Table 3:** Summary of logistic regression of scoring systems and ratios for predicting exacerbations.

Model	n	Events	OR (95% CI)	β	SE	z-Value	*p*-Value	AIC	AUC (95% CI)	AUC (Optimism-Corrected 95% CI)	Optimism
log (CRP)	131	75	2.23 (1.32–3.95)	0.80	0.28	2.9	0.004	143.8	0.865 (0.803–0.926)	0.807 (0.788–0.851)	0.057
Albumin	131	75	0.35 (0.06–1.69)	−1.05	0.84	−1.27	0.206	151.4	0.838 (0.771–0.905)	0.774 (0.772–0.827)	0.063
CRP/albumin	131	75	1.08 (1.03–1.15)	0.08	0.03	2.76	0.006	140.1	0.868 (0.808–0.929)	0.814 (0.800–0.860)	0.055
LMR	131	75	0.51 (0.29–0.87)	−0.67	0.28	−2.37	0.018	146.3	0.846 (0.782–0.911)	0.786 (0.784–0.839)	0.061
NLR	131	75	1.52 (1.12–2.21)	0.42	0.17	2.44	0.015	145	0.855 (0.792–0.917)	0.796 (0.792–0.843)	0.058
GPS	131	75	2.75 (1.10–7.57)	1.01	0.49	2.07	0.038	148.4	0.846 (0.778–0.914)	0.786 (0.781–0.839)	0.060
hs-GPS	131	75	2.17 (0.89–5.50)	0.77	0.46	1.68	0.093	150.1	0.840 (0.774–0.906)	0.777 (0.778–0.830)	0.063

CRP, C-reactive protein; LMR, lymphocyte–monocyte ratio; NLR, neurophil–lymphocyte ratio; GPS, Glasgow Prognostic Score; hs-GPS, high-sensitivity Glasgow Prognostic Score; CI, confidence interval; AUC, area under the curve; AIC, Akaike information criterion; SE, standard error.

**Table 4:** Diagnostic performance of biomarkers at the optimal threshold for predicting exacerbations.

Model	Threshold	Sensitivity	Specificity	PPV (95% CI)	NPV (95% CI)
CRP(log)	0.53	0.84	0.77	0.829 (0.73–0.90	0.78 (0.66–0.87)
Albumin	0.31	0.97	0.54	0.74 (0.64–0.81)	0.94 (0.80–0.98)
CRP/albumin	0.42	0.91	0.68	0.79 (0.69–0.86)	0.84 (0.71–0.92)
LMR	0.43	0.87	0.66	0.77 (0.67–0.85)	0.79 (0.65–0.88)
NLR	0.38	0.92	0.59	0.75 (0.65–0.83)	0.85 (0.70–0.93)
GPS	0.42	0.91	0.70	0.80 (0.70–0.87)	0.85 (0.72–0.92)
hs-GPS	0.39	0.91	0.64	0.77 (0.67–0.85)	0.84 (0.70–0.92)

CRP, C-reactive protein; LMR, lymphocyte–monocyte ratio; NLR, neurophil–lymphocyte ratio; GPS, Glasgow Prognostic Score; hs-GPS, high-sensitivity Glasgow Prognostic Score; PPV, positive predictive value; NPV, negative predictive value; CI, confidence interval.

## Data Availability

All data can be presented on request.

## Supplementary Materials

The following supporting information can be downloaded at: https://www.mdpi.com/article/10.3390/diagnostics15212761/s1; Table S1: Adjusted logistic regression results for individual biomarkers and scoring systems predicting future exacerbations; Table S2: Logistic regression of scoring systems and ratios predicting acute exacerbations, stratified by absence of baseline exacerbations; Table S3: Diagnostic performance of biomarkers at the optimal threshold for predicting exacerbations, stratified by absence of baseline exacerbations; Table S4: Logistic regression of scoring systems and ratios predicting acute exacerbations, stratified by presence of baseline exacerbations; Table S5: Diagnostic performance of biomarkers at the optimal threshold for predicting exacerbations, stratified by presence of baseline exacerbations; Table S6: Logistic regression of scoring systems and ratios predicting acute exacerbations, excluding pwCF treated with CFTR modulators; Table S7: Diagnostic performance of biomarkers at the optimal threshold for predicting exacerbations, excluding pwCF treated with CFTR modulators.

## Abbreviations

AIC: Akaike information criterion; AUC: area under the curve; BAL: bronchoalveolar lavage; BMI: body mass index; CF: cystic fibrosis; CFTR: cystic fibrosis transmembrane conductance regulator; CPC-M: Comprehensive Pneumology Center Munich; DZL: German Center for Lung Research; FEV1: forced expiratory volume in 1 s; FU: follow-up (period); GPS: Glasgow Prognostic Score; hs-GPS: high-sensitivity modified Glasgow Prognostic Score; IQR: interquartile range; LMR: lymphocyte-to-monocyte ratio; LMU: Ludwig Maximilian University (Munich); NLR: neutrophil-to-lymphocyte ratio; OR: odds ratio; PA: Pseudomonas aeruginosa; PEx: pulmonary exacerbation(s); ppFEV1: percent predicted FEV1; pwCF: people with cystic fibrosis; SD: standard deviation.
