# Supplementary material for: Predicting Pulmonary Exacerbations in Cystic Fibrosis Using Inflammation-Based Scoring Systems
Source: Diagnostics (Basel). 2025 Oct 31;15(21):2761. doi: 10.3390/diagnostics15212761 (PMC12610019; doi:10.3390/diagnostics15212761)
Supplement: Supplementary file 1 [file diagnostics-15-02761-s001.zip › diagnostics-3851652-supplementary.pdf]

Table S1. Adjusted logistic regression results for individual biomarkers and scoring systems predicting future exacerbations.

Model	Coefficient	n	events	OR (95% CI)	SE	z	p
CRP(log)	(Intercept)	131	75	49.70 (0.16–21530.64)	2.98	1.31	0.19
	Age	131	75	1.03 (0.97–1.09)	0.03	1.02	0.31
	Sex	131	75	1.02 (0.34–3.03)	0.55	0.03	0.98
	BMI at baseline	131	75	0.70 (0.52–0.92)	0.15	-2.41	0.02
	Diabetes mellitus	131	75	2.67 (0.95–8.05)	0.54	1.81	0.07
	delF508 homocygote	131	75	0.81 (0.30–2.13)	0.50	-0.43	0.66
	Exazerbations during baseline	131	75	3.70 (1.37–10.60)	0.52	2.53	0.01
	CTFR modulator therapy	131	75	1.07 (0.22–5.93)	0.83	0.08	0.93
	Pseudomonas aeruginosa	131	75	2.71 (0.76–10.28)	0.66	1.52	0.13
	Stenotrophomonas maltophilia	131	75	3.94 (0.99–17.52)	0.72	1.9	0.06
	Mycobacterium abscessus	131	75	3.01 (0.29–70.83)	1.29	0.86	0.39
	Achromobacter xylosoxidans	131	75	3.83 (0.80–26.02)	0.86	1.57	0.12
	CRP(log)/Albumin(adjusted)	131	75	1.08 (1.03–1.15)	0.03	2.76	0.01
Albumin	(Intercept)	131	75	1654.13 (0.42–12981672.75)	4.35	1.71	0.09
	Age	131	75	1.01 (0.95–1.06)	0.03	0.21	0.84
	Sex	131	75	0.94 (0.33–2.62)	0.52	-0.13	0.90
	BMI at baseline	131	75	0.80 (0.62–1.00)	0.12	-1.92	0.05
	Diabetes mellitus	131	75	1.78 (0.67–4.90)	0.51	1.14	0.26
	delF508 homocygote	131	75	0.82 (0.32–2.07)	0.47	-0.42	0.67
	Exazerbations during baseline	131	75	5.80 (2.32–15.62)	0.48	3.64	0.00
	CTFR modulator therapy	131	75	2.22 (0.48–12.11)	0.81	0.98	0.33
	Pseudomonas aeruginosa	131	75	2.84 (0.88–9.82)	0.61	1.71	0.09
	Stenotrophomonas maltophilia	131	75	4.54 (1.33–17.87)	0.66	2.31	0.02
	Mycobacterium abscessus	131	75	2.90 (0.32–65.16)	1.24	0.86	0.39
	Achromobacter xylosoxidans	131	75	2.88 (0.65–17.16)	0.81	1.3	0.19

	Albumin(adjusted)	131	75	0.35 (0.06–1.69)	0.84	-1.27	0.21
LMR	(Intercept)	131	75	98.14 (0.52–29376.60)	2.76	1.66	0.10
	Age	131	75	0.99 (0.93–1.05)	0.03	-0.23	0.82
	Sex	131	75	0.68 (0.23–1.96)	0.55	-0.71	0.48
	BMI at baseline	131	75	0.82 (0.64–1.03)	0.12	-1.61	0.11
	Diabetes mellitus	131	75	2.05 (0.75–5.92)	0.52	1.38	0.17
	delF508 homocygote	131	75	0.77 (0.30–1.99)	0.48	-0.53	0.59
	Exazerbations during baseline	131	75	5.76 (2.29–15.70)	0.49	3.59	0.00
	CTFR modulator therapy	131	75	2.11 (0.46–11.27)	0.80	0.93	0.35
	Pseudomonas aeruginosa	131	75	3.53 (1.08–12.78)	0.62	2.02	0.04
	Stenotrophomonas maltophilia	131	75	5.97 (1.68–25.15)	0.68	2.61	0.01
	Mycobacterium abscessus	131	75	1.38 (0.15–31.14)	1.25	0.25	0.80
	Achromobacter xylosoxidans	131	75	2.64 (0.58–16.04)	0.83	1.18	0.24
	LMR(adjusted)	131	75	0.51 (0.29–0.87)	0.28	-2.37	0.02
NLR	(Intercept)	131	75	2.94 (0.02–696.79)	2.70	0.4	0.69
	Age	131	75	0.99 (0.94–1.05)	0.03	-0.23	0.82
	Sex	131	75	0.91 (0.31–2.61)	0.54	-0.18	0.86
	BMI at baseline	131	75	0.82 (0.64–1.03)	0.12	-1.63	0.10
	Diabetes mellitus	131	75	1.96 (0.72–5.57)	0.52	1.31	0.19
	delF508 homocygote	131	75	0.78 (0.30–2.02)	0.48	-0.51	0.61
	Exazerbations during baseline	131	75	5.73 (2.23–15.99)	0.50	3.51	0.00
	CTFR modulator therapy	131	75	1.65 (0.33–9.29)	0.84	0.6	0.55
	Pseudomonas aeruginosa	131	75	3.55 (1.07–13.12)	0.63	2	0.05
	Stenotrophomonas maltophilia	131	75	3.35 (0.93–13.52)	0.67	1.8	0.07
	Mycobacterium abscessus	131	75	1.31 (0.14–29.61)	1.25	0.22	0.83
	Achromobacter xylosoxidans	131	75	1.87 (0.39–11.42)	0.84	0.75	0.46
	NLR(adjusted)	131	75	1.52 (1.12–2.21)	0.17	2.44	0.01

GPS	(Intercept)	131	75	29.29 (0.15–8308.54)	2.76	1.22	0.22
	Age	131	75	1.02 (0.96–1.08)	0.03	0.62	0.54
	Sex	131	75	0.93 (0.32–2.65)	0.53	-0.14	0.89
	BMI at baseline	131	75	0.76 (0.58–0.96)	0.13	-2.17	0.03
	Diabetes mellitus	131	75	2.05 (0.76–5.72)	0.51	1.4	0.16
	delF508 homocygote	131	75	0.78 (0.30–1.99)	0.48	-0.52	0.61
	Exazerbations during baseline	131	75	4.61 (1.77–12.85)	0.50	3.05	0.00
	CTFR modulator therapy	131	75	1.57 (0.35–8.31)	0.80	0.57	0.57
	Pseudomonas aeruginosa	131	75	3.02 (0.92–10.73)	0.62	1.78	0.07
	Stenotrophomonas maltophilia	131	75	4.01 (1.11–16.39)	0.68	2.05	0.04
	Mycobacterium abscessus	131	75	2.32 (0.24–53.84)	1.27	0.66	0.51
	Achromobacter xylosoxidans	131	75	3.16 (0.69–20.22)	0.84	1.37	0.17
	GPS(adjusted)	131	75	2.75 (1.10–7.57)	0.49	2.07	0.04

Hs-GPS	(Intercept)	131	75	18.71 (0.11–4117.45)	2.65	1.11	0.27
	Age	131	75	1.02 (0.96–1.08)	0.03	0.62	0.54
	Sex	131	75	0.93 (0.32–2.64)	0.53	-0.13	0.90
	BMI at baseline	131	75	0.76 (0.59–0.96)	0.12	-2.2	0.03
	Diabetes mellitus	131	75	2.11 (0.79–5.91)	0.51	1.47	0.14
	delF508 homocygote	131	75	0.86 (0.34–2.19)	0.48	-0.32	0.75
	Exazerbations during baseline	131	75	5.16 (2.02–14.14)	0.49	3.33	0.00
	CTFR modulator therapy	131	75	1.73 (0.37–9.54)	0.82	0.67	0.50
	Pseudomonas aeruginosa	131	75	2.68 (0.82–9.41)	0.62	1.6	0.11
	Stenotrophomonas maltophilia	131	75	4.32 (1.22–17.74)	0.67	2.17	0.03
	Mycobacterium abscessus	131	75	3.36 (0.36–77.86)	1.26	0.96	0.34
	Achromobacter xylosoxidans	131	75	3.06 (0.65–19.41)	0.84	1.33	0.18
	hsGPS(adjusted)	131	75	2.17 (0.89–5.50)	0.46	1.68	0.09

CRP, C-reactive protein; LMR, Lymphocyte-monocyte ratio; NLR, Neurophil-lymphocyte ratio; GPS, Glasgow Prognostic Score; hs-GPS, High-sensitive Glasgow Prognostic Score; CI, confidence interval; AUC, Area under the curve; AIC, Akaike information criterion; SE, Standard Error; BMI, Body mass index; CFTR , cystic fibrosis transmembrane conductance regulator

Table S2: Summary of Logistic Regression of Scoring Systems and Ratios for predicting acute exacerbations, stratified by the absence of exacerbations during the baseline period.

Model	N	Events	OR (95% CI)	β	SE	z-value	p-value	AIC	AUC (95% CI)	AUC (optimism-corrected 95% CI)	Optimism
CRP(log)	67	23	2.99 (1.25–8.65)	1.10	0.48	2.27	0.02	82.6	0.85 (0.76–0.94)	0.73 (0.70–0.85)	0.12
Albumin	67	23	0.73 (0.05–9.72)	-0.31	1.29	-0.24	0.81	88.8	0.82 (0.72–0.92)	0.69 (0.68–0.82)	0.13
CRP/Albumin	67	23	1.22 (1.07–1.46)	0.20	0.08	2.61	0.01	76.6	0.89 (0.81–0.97)	0.78 (0.74–0.87)	0.11
LMR	67	23	0.48 (0.20–0.96)	-0.74	0.41	-1.8	0.07	84.4	0.83 (0.73–0.93)	0.70 (0.70–0.83)	0.12
NLR	67	23	1.56 (1.01–2.58)	0.44	0.23	1.89	0.06	84.7	0.84 (0.75–0.94)	0.72 (0.70–0.84)	0.12
GPS	67	23	26.93 (3.49–417.97)	3.29	1.18	2.78	0.01	77.6	0.90 (0.83–0.98)	0.79 (0.71–0.88)	0.11
hs-GPS	67	23	1.48 (0.41–6.14)	0.39	0.68	0.58	0.56	88.5	0.83 (0.73–0.93)	0.70 (0.68–0.83)	0.13

CRP, C-reactive protein; LMR, Lymphocyte-monocyte ratio; NLR, Neurophil-lymphocyte ratio; GPS, Glasgow Prognostic Score; hs-GPS, High-sensitive Glasgow Prognostic Score; CI, confidence interval; AUC, Area under the curve; AIC, Akaike information criterion; SE, Standard Error

Table S3: Diagnostic performance of biomarkers at the optimal threshold for predicting exacerbations, stratified by the absence of exacerbations during the baseline period.

Model	Threshold	Sensitivity	Specificity	PPV	NPV
CRP(log)	0.27	0.96	0.71	0.63	0.97
Albumin	0.33	0.83	0.75	0.63	0.89
CRP/Albumin	0.30	0.96	0.68	0.61	0.97
NLR	0.30	0.96	0.73	0.65	0.97
LMR	0.33	0.91	0.75	0.66	0.94
GPS	0.43	0.91	0.86	0.78	0.95
hs-GPS	0.32	0.91	0.73	0.64	0.94

CRP, C-reactive protein; LMR, Lymphocyte-monocyte ratio; NLR, Neurophil-lymphocyte ratio; GPS, Glasgow Prognostic Score; hs-GPS, High-sensitive Glasgow Prognostic Score; PPV, positive predictive value; NPV, negative predictive value; CI, confidence interval

Table S4: Summary of Logistic Regression of Scoring Systems and Ratios for predicting acute exacerbations, stratified by the presence of an exacerbation during the baseline period.

Model	N	Events	OR (95% CI)	β	SE	z-value	p-value	AIC	AUC (95% CI)	AUC (optimism-corrected 95% CI)	Optimism
CRP(log)	64	52	1.93 (0.82–5.30)	0.66	0.46	1.42	0.16	71.40	0.84 (0.74–0.94)	0.66 (0.64–0.82)	0.17
Albumin	64	52	0.20 (0.01–2.93)	-1.63	1.52	-1.07	0.28	72.30	0.82 (0.69–0.94)	0.65 (0.63–0.81)	0.17
CAP (CRP/Albumin)	64	52	1.04 (1.00–1.13)	0.04	0.03	1.31	0.19	70.60	0.83 (0.72–0.93)	0.66 (0.64–0.82)	0.17
LMR	64	52	0.61 (0.24–1.44)	-0.49	0.45	-1.09	0.28	72.40	0.82 (0.70–0.94)	0.65 (0.62–0.80)	0.17
NLR	64	52	1.96 (1.06–5.41)	0.67	0.41	1.64	0.10	68.70	0.82 (0.72–0.93)	0.66 (0.67–0.83)	0.17
GPS	64	52	0.89 (0.24–3.33)	-0.11	0.65	-0.17	0.86	73.60	0.80 (0.66–0.95)	0.63 (0.61–0.79)	0.18
hs-GPS	64	52	3.59 (0.71–21.60)	1.28	0.85	1.50	0.13	71.30	0.85 (0.75–0.95)	0.68 (0.63–0.83)	0.17

CRP, C-reactive protein; LMR, Lymphocyte-monocyte ratio; NLR, Neurophil-lymphocyte ratio; GPS, Glasgow Prognostic Score; hs-GPS, High-sensitive Glasgow Prognostic Score; CI, confidence interval; AUC, Area under the curve; AIC, Akaike information criterion; SE, Standard Error

Table S5: Diagnostic performance of biomarkers at the optimal threshold for predicting exacerbations, stratified by the presence of an exacerbation during the baseline period.

Model	Threshold	Sensitivity	Specificity	PPV	NPV
CRP(log)	0.79	0.75	0.92	0.98	0.46
Albumin	0.72	0.81	0.83	0.96	0.50
CRP/Albumin	0.78	0.73	0.92	0.97	0.44
NLR	0.82	0.65	1.00	1.00	0.40
LMR	0.71	0.81	0.75	0.93	0.47

GPS	0.81	0.71	0.83	0.95	0.40
hs-GPS	0.76	0.83	0.83	0.96	0.53

CRP, C-reactive protein; LMR, Lymphocyte-monocyte ratio; NLR, Neurophil-lymphocyte ratio; GPS, Glasgow Prognostic Score; hs-GPS, High-sensitive Glasgow Prognostic Score; PPV, positive predictive value; NPV, negative predictive value; CI, confidence interval

Table S6: Summary of Logistic Regression of Scoring Systems and Ratios for predicting acute exacerbations, excluding pwCF who were treated with CFTR modulators.

Model	N	Events	OR (95% CI)	β	SE	z-value	p-value	AIC	AUC (95% CI)	AUC (optimism-corrected 95% CI)	Optimism
log(CRP)	111	59	1.86 (1.08–3.33)	0.62	0.28	2.18	0.029	130.8	0.85 (0.77–0.92)	0.77 (0.77–0.84)	0.07
Albumin	111	59	0.33 (0.06–1.70)	-1.10	0.87	-1.27	0.203	134.2	0.83 (0.76–0.90)	0.75 (0.76–0.82)	0.08
CRP/Albumin	111	59	1.06 (1.02–1.14)	0.06	0.03	2.23	0.026	127.4	0.85 (0.78–0.92)	0.79 (0.78–0.85)	0.07
LMR	111	59	0.54 (0.30–0.92)	-0.62	0.29	-2.13	0.033	130.3	0.83 (0.76–0.91)	0.76 (0.77–0.83)	0.07
NLR	111	59	1.40 (1.03–2.03)	0.34	0.17	1.96	0.051	131.1	0.84 (0.77–0.91)	0.77 (0.77–0.83)	0.07
GPS	111	59	2.03 (0.77–5.80)	0.71	0.51	1.40	0.163	133.8	0.83 (0.75–0.91)	0.76 (0.76–0.83)	0.07
Hs-GPS	111	59	1.79 (0.73–4.61)	0.58	0.47	1.25	0.211	134.3	0.83 (0.75–0.90)	0.75 (0.75–0.82)	0.08

CRP, C-reactive protein; LMR, Lymphocyte-monocyte ratio; NLR, Neurophil-lymphocyte ratio; GPS, Glasgow Prognostic Score; hs-GPS, High-sensitive Glasgow Prognostic Score; CI, confidence interval; AUC, Area under the curve; AIC, Akaike information criterion; SE, Standard Error; pwCF, people with Cystic Fibrosis; CFTR, Cystic Fibrosis Transmembrane Conductance Regulator

Table S7: Diagnostic performance of biomarkers at the optimal threshold for predicting exacerbations, excluding pwCF who were treated with CFTR modulators.

Model	Threshold	Sensitivity	Specificity	PPV	NPV
CRP(log)	0.4169	0.898	0.692	0.768	0.857
Albumin	0.4235	0.864	0.654	0.739	0.81
CRP/Albumin	0.4159	0.932	0.692	0.775	0.9
NLR	0.5264	0.763	0.769	0.789	0.741
LMR	0.6339	0.678	0.827	0.816	0.694
GPS	0.4763	0.847	0.731	0.781	0.809
Hs-GPS	0.4022	0.898	0.654	0.746	0.85

CRP, C-reactive protein; LMR, Lymphocyte-monocyte ratio; NLR, Neurophil-lymphocyte ratio; GPS, Glasgow Prognostic Score; hs-GPS, High-sensitive Glasgow Prognostic Score; PPV, positive predictive value; NPV, negative predictive value; CI, confidence interval; pwCF, people with Cystic Fibrosis; CFTR, Cystic Fibrosis Transmembrane Conductance Regulator
